# Macromolecular Crowding and DNA: Bridging the Gap between In Vitro and In Vivo

**DOI:** 10.3390/ijms242417502

**Published:** 2023-12-15

**Authors:** Dylan Collette, David Dunlap, Laura Finzi

**Affiliations:** Department of Physics, College of Arts & Sciences, Emory University, Atlanta, GA 30322, USA; dcollette@oglethorpe.edu (D.C.); ddunlap@emory.edu (D.D.)

**Keywords:** macromolecular crowding, DNA, polyethylene glycol (PEG), dextran, bovine serum albumin (BSA), liquid–liquid phase separation (LLPS)

## Abstract

The cellular environment is highly crowded, with up to 40% of the volume fraction of the cell occupied by various macromolecules. Most laboratory experiments take place in dilute buffer solutions; by adding various synthetic or organic macromolecules, researchers have begun to bridge the gap between in vitro and in vivo measurements. This is a review of the reported effects of macromolecular crowding on the compaction and extension of DNA, the effect of macromolecular crowding on DNA kinetics, and protein-DNA interactions. Theoretical models related to macromolecular crowding and DNA are briefly reviewed. Gaps in the literature, including the use of biologically relevant crowders, simultaneous use of multi-sized crowders, empirical connections between macromolecular crowding and liquid–liquid phase separation of nucleic materials are discussed.

## 1. Introduction

Much experimental effort is devoted to mimicking cellular conditions in vitro. However, the complexity of living cells makes emulating cellular environments quite difficult. Biological cells are highly crowded with the cytoskeleton, dissolved salts, proteins, and nucleic acids of various sizes and structures [1]. This crowded environment shapes a variety of biological processes and functions, including viral infection, gene expression, chromosomal compaction, replication, and transcription [2,3,4,5,6]. The full extent of the impact that crowding has on the intracellular dynamics of nucleic acids remains a topic of debate. The average volume fraction of a cell that is taken up by solute molecules is 30%, although this value may reach 40% [3,4,6,7,8]. This cytoplasm is delimited by the cellular membrane to a few cubic micrometers for bacteria, and approximately one hundred cubic micrometers for eukaryotic cells [3]. Additionally, high compartmentalization is achieved within cells, even in the absence of membrane-enclosed organelles, leading to local concentrations of specific molecules which can further enhance crowding [9]. Crowding created by membrane confinement has proven difficult to reproduce in vitro and is often neglected in experiments. Nonetheless, a variety of small molecules contributing to crowding have been experimentally investigated. This review examines the effect of macromolecules, or polymers, on nucleic acids and their transactions, which is highly relevant to our understanding of genome structure and function and to the correlation of in vitro to in vivo observations [5,10,11,12]. A summary of the techniques most commonly used and of the effects observed is provided in Table 1 and Table 2, respectively.

## 2. Compaction and Extension of DNA

**Figure 1:**
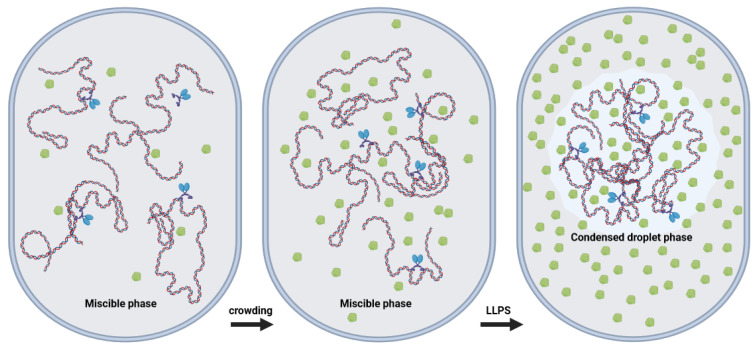
Illustration of DNA condensation due to macromolecular crowding within a cell or nucleus. (**left**) Under low crowding conditions, a miscible phase contains the homogeneous mixture of non-specific crowders (green), non-specific, mostly intramolecular crosslinkers (blue), and DNA molecules. (**center**). As the concentration of crowders increases, depletion forces rise and DNA molecules condense, which enhances crosslinker affinity and intermolecular crosslinks. (**right**). At even higher crowder concentrations, DNA condenses further, stabilized by transient intermolecular crosslinks, in a droplet phase with no membrane boundary, producing a separate liquid phase (liquid–liquid phase separation (LLPS). Created with Biorender.com.

An *Escherichia coli* (*E. coli*) cell, to consider for example a commonly studied bacterium and model system, is around one micron in length, while the length of the DNA compacted inside is over a millimeter [66] and the ensemble of all solutes constitute 30–40% of the intracellular volume [3,4,6,7,8,67]. The relationship between molecular crowding and DNA compaction into more dense structures such as rods, fibers, flexible rings, toroids, and hierarchical coils [68] is of great interest to understand the forces driving DNA packaging and architecture. The compaction/decompaction of DNA plays a key role for its organization within the cell and is a fundamental for the storage, maintenance and processing of genetic information. In prokaryotes, this regulation involves the interplay of DNA supercoiling [69], macromolecular crowding, and nucleoid-associated proteins (NAPs) [42,70,71,72,73,74]. In general, a variety of agents such as multivalent cations [75,76,77], cationic lipids [78], detergents [79], peptides [80], and many non-interacting polymers [81,82], may induce the collapse of DNA chains (Figure 1) which has application in packaging for gene therapy. Tuning the condensation/decondensation equilibrium of DNA is also fundamental to regulate access to the double helix and thereby gene regulation. The following subsections include descriptions of experiments with three commonly used macromolecular crowders: polyethylene glycol, dextran and bovine serum albumin.

### 2.1. Polyethylene Glycol

Polyethylene glycol (PEG) is a neutral polymer with industrial and medical uses. This polyether compound, derived from petroleum, has been widely used in medicine due to its biocompatibility, non-toxic features, and high solubility [83,84]. A prominent use of PEG is as a DNA carrier for therapeutic gene delivery [85]. In the presence of salts, PEG can induce DNA condensation through a process commonly referred to as polymer and salt-induced (psi, Ψ) condensation [86,87], which was first described by Lerman in 1971 [82]. Since then, there have been many experimental studies of Ψ condensation [47,49,50,51,52,53,54,55,56,57,58,59]. Vasilevskaya et al. investigated the compaction of a single DNA molecule in a PEG solution via fluorescence microscopy [56]. They found that the critical concentration of PEG required to induce DNA compaction decreases with increasing degree of PEG polymerization and salt concentration. This was interpreted as evidence that the contacts between DNA and PEG are thermodynamically unfavorable. Thus, the addition of PEG makes a poorer quality solution for DNA and favors intersegmental interactions between DNA molecules.

This effect was confirmed by Ramos et al. [47] using circular dichroism (CD) spectroscopy. These authors also reported that conditions of intermolecular condensation (aggregates of 4 kbp linear DNA) were very similar to those observed previously for intramolecular condensation [60,86]. Similarities between inter- or intramolecular DNA condensates extended to resolubilization by further addition of neutral polymer [56] and strong dependence of very dilute solutions of 169 kbp DNA on the concentrations of PEG and salt [56,57]. Using a 40 mM DNA solution in 0.2 M NaCl with varied amounts (*w*/*w*) of PEG 2000 (2000 indicates the average molecular weight in Da), Ramos et al. [47] found that subtle changes of PEG concentration from 19% to 20% significantly changed the CD spectrum, displacing a broad negative band from 240 to 270 nm and a positive band from 275 to above 300 nm, as is characteristic for Ψ condensation [48]. Continued addition of PEG up to 22% restored the typical B-form DNA spectrum, producing what they referred to as ‘reentrant decondensation’. The authors concluded that suprathreshold concentrations of PEG disfavor Ψ condensation leaving DNA dispersed in solution. Measurements in which the salt concentration was varied at a fixed concentration of PEG yielded the same results, suggesting thermodynamic equilibrium.

The effect of monovalent versus divalent cations on DNA condensation by crowders was examined by Cheng et al. Using magnetic tweezers, these authors showed that condensation was induced by PEG of varying molecular weights (PEG 600 and PEG 6000) in the presence of NaCl, as well as divalent MgCl^2^ [17]. Interestingly, they found that the critical condensation force (the tension below which DNA transitions from a chain to a globular state) for DNA in a 30% volume fraction solution of PEG increased linearly with NaCl concentration. Higher molecular weight PEG also increased the value of the critical condensation force. In contrast, the critical condensation force as a function of increasing MgCl^2^ concentration was parabolic. Furthermore, while DNA condensation in 100 mM Tris-HCl buffer required at least 300 mM Na^+^, PEG 6000 alone could induce DNA condensation in the absence of salt. By varying PEG 6000 concentration, a decrease in the critical force was observed, below which condensation spontaneously occurred from ∼0.6 pN at 30% PEG concentration to ∼0.1 pN at 18% PEG concentration. There was no detectable condensation in solutions with concentrations of PEG 6000 under 18%. Atomic force micrographs of the compact DNA–PEG complexes confirmed the salt dependence observed with magnetic tweezers. Thus, cations, Na^+^ or Mg^2+^ play an important role in Ψ condensation and their valency affects the nature and stability of the condensates.

How crowding affects the torsional state of DNA was investigated by Scott et al., who conducted single molecule Convex Lens-induced Confinement (CLiC) experiments. The authors indirectly measured DNA unwinding of the Site 1 region of pUC19 by the binding of a complementary fluorescently labeled oligo. They used 8 kDa PEG (PEG 8000), 10 or 55 kDa polyvinylpyrrolidone (PVP) at 12.5% *w/v* in a buffer with an ionic strength of 150 mM (137.4 mM NaCl, 12 mM Tris, 25 mM HEPES, pH 8.0) at 37 °C. DNA unwinding was recorded to increase with increasing concentrations of 8 kDa PEG over a range of 0 to 20% (*w*/*v*). This observation was confirmed even with increasing salt concentration, which was varied over a range of 22.5–150 mM. Earlier theoretical work had demonstrated that increasing salt decreases unwinding [88] and the effective diameter of the double helix [89]. Using a second-order reaction model, binding rates and the unwound state of plasmids were estimated, assuming the rates of unwinding and rewinding were negligible compared to the rate of binding [64].

The effect of crowder structure on DNA condensation was investigated by Mardoum et al. [2]. Examining DNA conformation and diffusion as a function of crowder structure, they found that branched, rigid crowders such as PEG and Ficoll compact DNA, while linear, flexible crowders, such as some molecular weights and preparations of dextran, caused DNA to extend. Although different crowder structures induced different DNA configurations, the extent to which increasing crowder concentrations reduced DNA mobility was similar.

Crowding effects on DNA condensation by proteins was, instead, investigated by Cristofalo et al. using magnetic tweezers (MT), PEG 1500, and the nucleoid protein H-NS [42]. Force-extension curves in the presence of PEG were consistent with unraveling the DNA filament from a disordered, collapsed globule [90,91,92]. With 13–22% volume fractions of PEG, DNA was condensed under low (<1.2 pN) tension. The observed DNA extension was consistent with three distinct regimes. Under low tensions, slight extension was associated with elastic deformation of a prolate globule. At intermediate tensions, the ends of the polymer unraveled from the globule forming ‘tadpole’ configurations with increased entropy in the extended part of the chain (tail). Under high tension, the polymer fully extended. From these measurements, the authors concluded that there is an evident cooperative effect between H-NS activity and the depletion forces induced by PEG.

### 2.2. Dextran

Dextran, a polymer consisting of D-glucose units, is also commonly used in macromolecular crowding experiments. It can be either a complex branched or linear glucan, depending on the molecular weight and preparation [93,94,95]. Dextran has been used to mimic intracellular crowding of DNA [24,96,97] for in vitro experiments and compacts linear and plasmid DNA differently [25]. This is not surprising, given that crowding affects DNA writhe and the number of plectonemes in plasmids [25,26].

The effects of macromolecular crowders on the transport and conformational dynamics of large DNA molecules was investigated by single molecule fluorescence microscopy and particle tracking [2]. The displacement of the center of mass was measured, as well as the conformational size, shape, and fluctuations of 115 kbp DNA diffusing through solutions of various macromolecular crowders. Investigators examined the role of crowder structure and concentration, along with ionic conditions, on the diffusion and configurational dynamics of DNA molecules. Linear, flexible polymers, such as some dextrans, were found to cause DNA to elongate. DNA diffusivity was found to decrease with increasing dextran concentrations but less than expected based on the increasing viscosity of the crowding solutions [18,19]. Indeed, the measured diffusion coefficients followed a weaker scaling with viscosity than the expected classical Stokes–Einstein scaling $D∼\eta^{-1}$ of the diffusion coefficient, *D*, with viscosity, η. Below, we review studies of the effect of dextran on DNA plasmids.

Light scattering measurements of the radius of gyration, $R_{g}$, of 2675 bp-long double stranded plasmids in their supercoiled and linear configurations in the presence of macromolecular crowding [24] revealed that the supercoiled plasmid initially expands and subsequently compacts as the volume fraction of the crowder increases. The extent of the expansion was found to be highly dependent on the size of the dextran nanoparticle crowders, with the smallest particles exhibiting the largest effect. The linearized plasmid experienced monotonic compaction with increasing crowder volume fraction, and there was no peak in the radius of gyration observed. Another study, using slightly longer supercoiled plasmids, showed the presence of two compaction regimes for plasmid–dextran combinations: one characterized by normal diffusion, the other by sub-diffusion (particles moving more slowly due to intermittent trapping) [25]. Within these two regimes, the plasmid conformation was sensitive to the size of the crowder. The authors proposed a generalized scaling law of $R_{g}$,

$$R_{g}≅\xi \left(x\right)R_{g0}^{2/(1+x)}d^{2/(1+x)}\psi^{-1/(1+x)}$$

in which $R_{g0}$ is the radius of gyration of plasmids in the uncrowded environment, ξ(x) is a prefactor representing the deviation of plasmid conformation from the conformation without crowding, *d* is the size of the crowder, ψ is the volume fraction of the crowding agent, and *x* is the measure of the conformational geometry of the plasmids. This equation was derived by balancing the elastic pressure due to conformational changes in the plasmid and the osmotic pressure that arises from depletion forces due to the crowders.

Overall, there is substantial evidence that the molecular weight of dextran is a key parameter: relatively low molecular weights cause DNA to extend, but as molecular weight and volume fraction increase, DNA compacts. It is important to note that dextran can either take a linear or a complex branched form depending on molecular weight and preparation; this is often ignored, but can significantly change results. Branched crowders, such a PEG and ficoll (not discussed here), tend to induce compaction of DNA, while linear crowders, like dextran, initially cause DNA to extend, but as crowder concentration is further increased, will induce compaction. Dextran and other naturally occurring crowding agents are less commonly used than synthetic crowders, such as PEG, due to their higher cost.

### 2.3. Bovine Serum Albumin

Bovine serum albumin (BSA), a small, stable, and moderately non-reactive protein [98], with isoelectric point at pH = 5.1–5.5 [99] (although the ExPASy protparam tool indicates 5.8), is often used as protein supplement in cell culture media [100]. Krotova et al. studied the conformational properties of DNA in a salt solution of BSA. They found that BSA compacts DNA due to depletion effects and strong electrostatic repulsion between the negatively charged protein and DNA [20]. Additionally, Yoshikawa et al. reported three compaction regimes with increasing BSA concentration, coiled DNA, partially compacted DNA, and fully compacted DNA [21].

**Figure 2:**
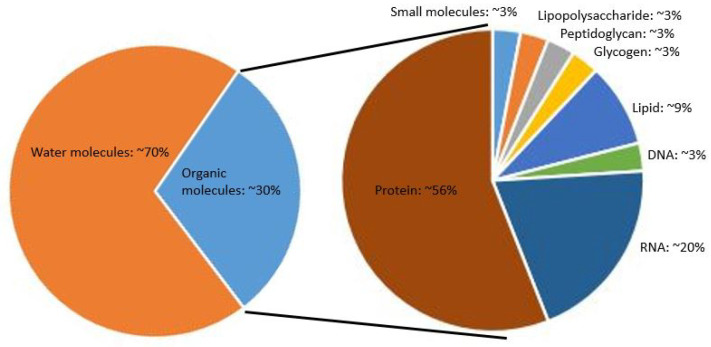
Molecular components of an *E. coli* cell [1].

The vast majority of publications investigating macromolecular crowding using BSA and other biologically relevant crowders focus on their effects on proteins rather than DNA (see, for example, [30]). It is clear that more work needs to be done to investigate biologically relevant macromolecular crowders acting on nucleic acids. Using RNA or various proteins with different biophysical characteristics is especially relevant because these macromolecules make up approximately 76% of the organic molecules inside a typical cell, as shown in Figure 2.

### 2.4. Outlook

Most investigations of macromolecular crowding of DNA employed non-physiological, albeit pharma-relevant, neutral crowders such as polyethylene glycol and dextran. These crowders are popular, because they are electrostatically neutral and commercially available in different degrees of polymerization (i.e., molecular weight). A systematic analysis of the effect of size, shape, and charge of crowders, in well-defined ionic conditions, on specific aspects of mechanical properties and biochemistry of DNA, including DNA topology, DNA-protein interactions, DNA repair, recombination, etc., is needed to better bridge the gap between in vitro and in vivo studies. In addition, a wider range of biologically relevant macromolecular crowders should be explored, including nucleic acids and cytoskeletal filaments normally present in the nucleus/nucleoid.

## 3. Kinetics

### 3.1. Crowders vs. Viscogens

The DNA conformation and condensation state greatly affect the activity of enzymes that process it. With up to 40% of the overall volume of the cytoplasm taken up by macromolecules [3,4,6,7,8,67], cells present a dense environment that likely changes the kinetics of biological reactions with respect to those observed in buffer [101,102,103,104]. In particular, the drastic increase in viscosity might slow macromolecular motions and kinetics [105,106].

For example, macromolecular crowding slows down the stochastic opening and closing of single-stranded DNA (ssDNA) hairpins and increases the steady-state fraction of closed hairpins significantly [22]. The authors, following the approach of Bonnet et al. [107] and Wallace et al. [36,108], and using a combination of fluorescence energy transfer and fluorescence correlation spectroscopy, observed the conformational fluctuations of an ssDNA hairpin. The kinetics of ssDNA constructs (beacons and controls) in water containing varying amounts of sucrose, or 200 Da-PEG (which is comparable in size to sucrose molecules) were compared with those observed in solutions containing 4 or 10 kDa PEG, or dextran. Increased concentrations of sucrose augment the viscosity of the fluid without producing sub-diffusion of tracer particles [40]. Acting as a pure viscogen, sucrose changed the opening and closing rates of the hairpin, but did not change the fraction of closed hairpins. The closing rate was approximated as a diffusive search of two binding partners separated by the length of the intervening ssDNA, while the opening rate was described by Kramer’s escape from a local potential minimum. In this analysis, the characteristic time scale of the conformational fluctuations of the hairpin decreased with sucrose concentration, while the steady-state fraction of closed hairpins remained constant, as observed experimentally. However, in the presence of high molecular weight PEG, or dextran, besides slower conformational fluctuations and significantly more closed hairpins were observed. Compared to the sucrose solution, PEG, and dextran increased the fraction of closed hairpins by about 16% and 70%, respectively, which was confirmed also by UV absorption measurements. Thus, Stiehl et al. concluded that biochemical reactions in crowded fluids are sensitive to both volume exclusion effects and changes in the diffusion characteristics of reactants due to changes in the viscoelastic properties of the fluid [22].

Macromolecular crowding could be responsible for the differences that are recorded between in vitro [37,109,110,111] and in vivo transcription data [65,112]. The effects of macromolecular crowding on transcription initiation by *E. coli* RNA polymerase (RNAP) have been investigated by Chung et al. [37], who found that large crowders affect initiation kinetics in ways apart from those expected from viscosity. Measurements were conducted using in vitro, quenching-based, single-molecule kinetic assays. The microviscosity experienced by RNAP-Promoter complexes was measured under various crowding conditions using fluorescence correlation spectroscopy (FCS), and all measurements were performed at 25 °C. They quantified the amount of transcript at time points when a reaction quencher was added [113] using ssDNA FRET probes complementary to the transcripts. Transcription initiation was tested in the presence of 25% glycerol, 15% PEG 8000, 15% Ficoll 70, and 5% Dextran 500 (*w/v*). Transcription initiation rates in the absence of viscogen/crowder were also measured for reference. Measurements revealed that rates of initiation in solutions containing PEG, Ficoll, or Dextran, were faster than in 25% glycerol, even though the viscosities of the polymer solutions were much higher than that of 25% glycerol. The effective viscosity of a crowded medium (the microviscosity) may differ from the bulk viscosity [114,115,116], and FCS showed that the microviscosity was much smaller than the bulk viscosity generated by large crowders, Ficoll 70 and Dextran 500, while micro and bulk viscosity due to Dextran 10 and PEG 8000 were comparable. A unidirectional first-order kinetics model was used to fit and extract kinetic rate constants. Since it has been demonstrated that the effect of viscosity on some protein folding kinetics follows Kramers theory [117,118,119], the extracted rates were adjusted using Kramers kinetic theory to account for viscosity effects and decouple them from volume exclusion effects [120]. The viscosity-adjusted kinetics rate constants showed acceleration by a factor of ∼2 for dextran 500 and ficoll 70, and ∼6 for PEG 8000, while the viscosity-adjusted rate constant for glycerol was only marginally affected. This reflects the fact that glycerol is only a viscogen, while large crowders affect transcription kinetics in other ways in addition to viscosity [37].

In summary, the size of crowders is a key factor. Larger molecular weights yield crowding effects such as caging or trapping due to entropic effects from volume exclusion, while smaller molecular weight crowders will intersperse with the DNA and increase the viscosity of the solution [121]. This is in agreement with the findings by Sozanski et al. [13] that small crowders stop the kinesin-1 motor at a viscosity of 5 mPa·s, while large crowders have no effect, even at much higher viscosities and indicates the general importance of the viscosity scaling paradigm [116,122,123] in nanomechanics.

### 3.2. Phase Separation

As far back as 1995, Walter and Brooks hypothesized that phase separation in the cytoplasm, due to macromolecular crowding, might be one basis for microcompartmentalization [14]. More recently, Levone et al. determined that the multifunctional DNA/RNA-binding protein fused in sarcoma (FUS), which is involved in splicing, translation, and mRNA transport [124], induces liquid–liquid phase separation (LLPS), and is also important for DNA repair initiation [15]. Their experiments definitively demonstrated the importance of LLPS. Shakya et al. investigated LLPS of histone proteins in regard to chromatin organization [125]. Histone proteins package cellular DNA into actively transcribed euchromatin domains, as well as suppressed heterochromatin domains. Through in cellulo and in vitro studies, they found that histones contribute to heterochromatin formation through reversible LLPS with DNA. In this case, liquid droplets form containing linker histome H1 and chromatin, and they likely govern the access of transcription factors and RNA to heterochromatin domains through charge balance, multicomponent interactions, and fluctuating levels of small molecules such as ATP. The tendency of H1 to form a separate phase was recently confirmed by the Liu lab using correlative fluorescence and optical tweezing [43]. A cartoon representation of macromolecular crowding-induced LLPS within a generic cell is shown in the third panel of Figure 1. Zhang and Kutateladze briefly summarized findings that LLPS is an intrinsic physicochemical property of chromatin [126].

The role of PEG in LLPS was investigated by Park et al. experimentally and with field-theory simulations via complex Langevin sampling that suggest PEG drives LLPS by dehydration of polymers [31]. The investigation focused on the coacervate phase, an aqueous phase rich in macromolecules such as synthetic polymers, proteins, or nucleic acids [127,128,129,130,131]. Complex coacervation (CC) is a phenomenon in which polyelectrolytes separate into a polyelectrolyte-dense phase and a polyelectrolyte-dilute phase [132]. CC is affected by a wide variety of parameters, such as ionic strength, pH, polyelectrolyte concentration, and molecular weight of the polyelectrolytes, as well as temperature [133].

PEG promotes phase separation to a higher extent than other inert polymers due to its spherical conformation [46]. This prompted the idea that crowding agents shift binding equilibria toward association and significantly extend the range of intracellular conditions under which interactions occur. Isothermal titration calorimetry and UV melting experiments indicated that crowding-induced effects are marginal under conditions that favor association of DNA strands, but become progressively larger when conditions deteriorate. Thus, in crowded environments, as discussed in Section 5, both entropic and enthalpic terms may favor aggregation: the first reducing excluded volume and the second increasing molecular interactions [97,102,134,135,136]. As a consequence, DNA packaging, association and aggregation of polymers, formation of tight oligomeric structures, and folding of extended polypeptides are significantly enhanced in the crowded cellular environment [137]. Most evidence suggests that crowding-mediated compaction stimulates association of biopolymers and modulation of reaction rates [16,38,39,97,135,136]. The thermal stability of both long and short dsDNA structures has been shown to increase in the presence of inert polymers such as PEG or dextran [46,61]. The addition of PEG raised the dsDNA melting point by 4 °C, and had similar results for dsDNA molecules containing one or two mismatched base pairs. Dextran 70 also increased the melting point for dsDNA, but not as effectively as PEG, causing only a 2 °C increase. In contrast to long DNA molecules that tend to undergo collapse and aggregation in the presence of PEG, short segments remain completely soluble. In the presence of inert polymers, the thermal stability of triple-stranded DNA is enhanced to a more significant extent than dsDNA molecules of comparable length [62,63]. Triplex stabilization was enhanced by both PEG and dextran 70, but significantly more so by PEG. Additionally, Goobes et al. observed that triplex motifs containing mismatched bases are also effectively stabilized by PEG; the magnitude of the PEG-mediated increase in the triplex melting temperature was found to be constant and independent of the number of mismatched bases in the triplex motif [46]. Throughout these measurements, various weights and concentrations of PEG were used. Concentrations were investigated over a range of 0–15%, and a linear increase in melting temperature was observed for both dsDNA and triplex-DNA. PEG samples with concentrations greater than 15% were not amenable to examination due to the increased viscosity. The correlation between the size of PEG polymers and the thermal stability of DNA species showed a slight increase from PEG 200 to PEG 1000, but the effects of size were negligible from PEG 1000 to PEG 8000.

A recent review explores how the selective interactions and specific functions of biomolecules exhibit temporal and spatial patterns in crowded environments of complex mixtures of biomolecules [1]. The properties driving these interactions have attracted considerable attention in recent years [138,139], and localization in LLPS droplets in the cytoplasm and nucleus is important for cellular regulation [139]. Membraneless organelles, also referred to as droplets, concentrates, or granules [140,141,142,143,144] are formed through reversible processes, which are sensitive to various external signals associated with cellular stress [131,145]. The stability of the phase-separated state must be due to enthalpic factors such as solute–solute interactions, which overcome the entropically favorable homogeneous single phase [1]. It has been previously shown that droplets can be formed by cationic peptides and mononucleotides in laboratory conditions [27]; additionally, droplets formed in cells often contain polyions such as cationic proteins and RNAs [146]. The absence of a boundary membrane enables water molecules and solutes to pass freely through the interface such that droplets can dynamically exchange contents with the surrounding environment [147,148]. Various droplets have been identified within cells, each serving a biological function. The nucleus alone contains the nucleolus [149], paraspeckles [150], nuclear speckles [141], Cajal bodies [142], and promyelocytic leukemia (PML) bodies [151]. These droplets perform many cellular functions, such as storing and regulating accessibility of RNA and transcription factors to regulate gene expression. The importance of LLPS and droplet formation within the highly crowded cellular environment highlights the need for systematic studies of the effects of macromolecular crowding on LLPS and droplet formation.

## 4. Protein–DNA Interactions

Macromolecular crowders are known to affect protein–DNA interactions through volume exclusion-derived phenomena such as trapping, caging, depletion, or electrostatic forces. For example, by influencing chromosome compaction through depletion-like interactions, as previously hypothesized [87,152], and later observed [153,154,155], macromolecular crowding affects chromosomal dynamics [156,157,158,159]. On the other hand, nucleoid-associated proteins (NAPs) can induce DNA condensation and modify chromosome organization by directly binding DNA [44,160,161,162]. NAPs recognize binding sites of 10–30 bps with different levels of specificity [163], and can cause bending, bridging, or wrapping of the double helix. Many NAPs remain bound for relatively long times [164,165,166,167]. Studies on NAPs and various concentrations of macromolecular crowders that do not bind or interact directly with DNA, such as PEG, have shown that there is a complex interplay between NAPs and crowders [32,45,49,51,56]. H-NS is a small (MW 15.5 kDa) NAP abundant in bacteria at approximately 20,000 molecules per cell (∼20 μM). It has a C-terminal DNA-binding domain and an N-terminal dimerization domain connected via a flexible linker [29,72,168,169,170]. It interacts with adjacent proteins bound to the same DNA molecule to stabilize DNA loops [169,171]. H-NS binds non-specifically with DNA, although it is known to favor AT-rich regions [172,173,174,175]. Cristofalo et al. used magnetic tweezers to observe the effects of PEG and externally applied tension on the interaction between DNA and H-NS. They found that compaction of DNA by H-NS and PEG is cooperative [42]. The experimental conditions used in these measurements were chosen to be similar to those found in the cellular environment, where the volume fraction of proteins is reported to be between 12 and 17% [165], and overall solute molecules constitute 30 to 40% [3,4,6,7,8,67].

Lin et al. used a different NAP, HU (Histone-like protein from strain U93) [41], which binds and compacts DNA. HU is highly expressed in most eubacteria with tens of thousands of copies per cell, and is one of the most abundant proteins in *E. coli* [176]. Both macromolecular crowding and salt conditions affected the binding of HU to DNA. At two different MgCl^2^ concentrations, three different crowders (blotting grade blocker (BGB), bovine serum albumin (BSA) and PEG 8000) were used in an effort to mimic the intracellular environment. BGB is a non-fat milk-product mixture comprised mostly of casein micelles containing large globular proteins ranging from 50 to 600 nm in diameter [28,177]. The presence of magnesium was dictated by its crucial role in the functionality of many proteins and enzymes [178,179,180,181,182,183]. Measurements were conducted using the tethered particle motion (TPM) technique, where DNA is attached to a substrate at one end, while the other is attached to an observable bead. The Brownian motion of the bead is monitored over time to reveal conformational changes in the DNA tether. PEG 8000 was not a useful crowding agent under these experimental conditions for concentrations above 9% (*w/v*) that produced adhesion between DNA, HU, and/or the glass surface of the microscope microchamber. PEG can be used at high crowder percentages in magnetic tweezers measurements, since tension on the DNA-tethered bead prevents sticking to the glass surface of the microscope microchamber [42].

Two HU binding regimes were observed, which were highly sensitive to crowding conditions. Magnesium ions enhanced the compaction of HU-DNA and suppressed filamentation, while BGB and BSA increased local concentrations of HU protein by more than 4-fold, and suppressed filament formation. In the absence of MgCl^2^, there were notable differences between 0.5% and 1% (*w/v*) BGB; maximal DNA compaction occurred with less HU in the lower, compared to the higher, BGB percentage (25 versus 100 nM HU). This observation implies a balance between crowding effects on binding energy and on diffusion; the authors suggested that too much crowding by BGB might hinder HU access to DNA. It was also observed that at higher HU concentrations in which HU forms filaments along the DNA, the root-mean-square (RMS) excursion of the tethered bead increased less as a function of BGB percentage, suggesting that BGB inhibits HU filamentation. In the presence of MgCl^2^, the RMS excursion decreased an additional 7% compared to the RMS excursion without BGB. Either BGB, or MgCl^2^, alone enhanced the compaction of DNA by HU and together produced additive effects.

BSA appeared to increase local HU concentrations. Three concentrations of BSA (1.25%, 5%, and 10% (*w/v*)) in the absence of MgCl^2^, and 10% BSA in the presence of MgCl^2^ were used to investigate the effects of this protein on HU binding. DNA compaction was observed with as little as 6.25 nM HU in 1.25% and 5% BSA, and 12.5 nM HU in 10% BSA, while it was not observed at concentrations lower than 150 nM HU without BSA. These observations suggest that BSA increases the local concentration of HU, and led Lin et al. to conclude that the most compact state attained in the presence of BSA does not differ significantly from that in the absence of BSA, since the minimum RMS excursion was approximately constant across measurements. However, too much BSA was less effective in promoting HU-driven DNA compaction [41]. Similarly to BGB, BSA might interfere with HU binding when present in high amounts, possibly interacting with HU and/or DNA [21], or causing steric effects. In the presence of MgCl^2^, the most compact state of the DNA occurred at a higher concentration of HU dimer. This observation cannot be explained by the impact of MgCl^2^ or BSA individually, but the authors noted that salt might alter the effective size of BSA, and therefore volume exclusion, to change the interaction of HU with DNA.

The impact of PEG 8000 on HU binding was also investigated with and without MgCl^2^. Unlike crowding with BGB or BSA, increasing percentages of PEG 8000 produced compaction at lower concentrations of HU, and it was suggested that MgCl^2^ works cooperatively with PEG 8000 to enhance DNA compaction [41].

## 5. Theoretical Models

**Figure 3:**
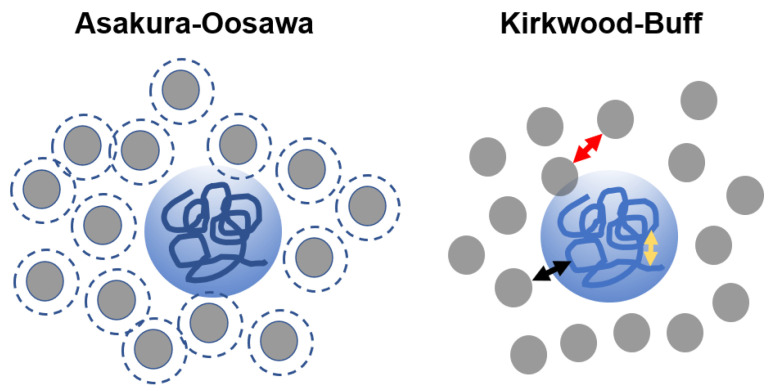
Schematic representation of the physical basis for the Asakura–Oosawa (AO; (**left**)) and the Kirkwood–Buff (KB; (**right**)) models. In the AO model, the molecules involved (here, DNA and crowder) are considered as hard spheres. The dashed line around each crowder “sphere” identifies the particle’s excluded volume. In the KB model, the arrows indicate the different types of molecular interactions (red: crowder–crowder; yellow: DNA–DNA; Black: crowder–DNA).

Theoretical models for crowding DNA polymers have assessed volume exclusion effects as a function of the relative size of crowders. The starting point of these models are most often the Asakura–Oosawa (AO) and the Kirkwood–Buff (KB) models. The AO model is used to describe the phase behavior of polymers and colloidal particles in a solvent. Here, polymers are treated as hard spheres that exclude each other and solvent molecules from the volume they occupy. This exclusion between particles leads to osmotic pressure (Π) and depletion forces, and the change in free energy imposed by the exclusion is ΔG = ΠΔ$V_{exclusion}$. As a coarse-grained model, the AO model captures the excluded volume effect among polymers and colloidal particles, but does not account for specific interactions, such as between polymer branches or between polymers and solvent molecules. The Kirkwood–Buff (KB) model, on the other hand, considers the spatial arrangement of polymer molecules and their specific interactions with other polymers and solvent molecules. The KB model calculates the chemical potential between two components in solution as the spatial integral over the pair distribution functions between the two components. At the simplest two-component system level with unbranched polymers, the KB model leads to the very same free energy as the AO model (Figure 3).

Cao et al. performed Langevin simulations to investigate the conformational change of a semi-flexible chain in a concentrated solution packed with spherical, active (self-motile) crowders [184]. They observed a novel shrinkage-to-swelling transition for polymers of low rigidity. A phase diagram was constructed in the parameter space of active force and size of crowders; the variation in the polymer radius of gyration demonstrated a non-monotonic dependence on the dynamic persistence path of the active particle. For small crowders, motile activity (force) increased the crowding-induced shrinkage of the chain, but as crowder size increased, it limited the crowding effect resulting in swelling of the polymer chains. For large crowders, the swelling effect from motile activity dominated the crowding effects.

The origin of the effective attractive interactions between and within macromolecules immersed in solutions containing cosolutes that are preferentially excluded from the macromolecular interfaces have been previously reviewed [185]. Although the effect of cosolutes is frequently excluded from molecular crowding investigations, using the Asakura–Oosawa model, based on completely entropic considerations, and Kirkwood–Buff solution theory, to alter the steric repulsion core with a ‘soft’ repulsive shell, adds an enthalpic contribution to the depletion force and suffices to rationalize the complete range of cosolute effects [186,187,188]. Considering that the cosolute–macromolecule interactions are temperature-dependent yields a depletion force that can be tuned to favor enthalpy over entropy. These simple considerations regarding the nature of the cosolute–macromolecule effective interaction help capture the essence of the effect of osmolytes.

The experimentally determined temperature dependence of protein stabilization in solutions crowded by preferentially excluded cosolutes was theoretically well-described by a model based on the Flory–Huggins approximation to regular solution theory. The model describes cosolutes in terms of their size, and two temperature-dependent microscopic parameters that correspond to cosolute–macromolecule and bulk solution interactions [186]. This model predicted a ‘depletion force’ that was able to account for experimentally observed stabilization in protein folding or association in the presence of excluded cosolutes. Additionally, the model predicts the full range of associated entropic and enthalpic components and depletion forces for specific cosolutes, in accordance with experiments. The depletion attraction that emerges is described by an effective, rather than molecular, volume, which results from the interplay between solvent, cosolute and macromolecular interactions. Examining the mean field theory of cosolute solutions in the limit of the Asakura and Oosawa model (AOM) [189,190], interactions of cosolutes with the surface of a macromolecule are purely steric and other interactions are omitted, assuming an ideal solution. For an ideal cosolute-solvent solution lacking nonideal mixing terms, the only remaining relevant parameters are the cosolute size and the parameters quantifying the cosolute interaction with the interface. This causes a deviation from the AOM prediction that is explained as due to an “effective” volume as opposed to the excluded volume.

Monte Carlo simulations by Shin et al. found that large crowders lead to caging of the polymer, while small polymers tend to mix with the chain monomers and increase the effective viscosity [121]. The focus of this study was to investigate the effects of volume fraction and crowder size on the kinetics of polymer coiling. By analyzing the coiling–uncoiling rates and coiling probabilities of the chain ends, it was shown that small crowders typically slow down the chain dynamics, while larger crowders seem to facilitate coiling. These observed effects were explained in terms of an effective solution viscosity and standard excluded volume; for small crowders, the effect of an increased viscosity dominates, while for larger crowders confinement effects drive dynamics.

## 6. Conclusions

Although the cellular environment is highly crowded, many laboratory measurements are conducted in dilute buffer. Introducing macromolecular crowders to laboratory settings is an important step towards bridging the gap between in vivo and in vitro measurements. This survey of the literature about the effects of macromolecular crowding on DNA structure and dynamics (Table 2) identifies three specific directions for further investigation. The first is using physiologically relevant crowders, such as RNA or proteins, as opposed to synthetic polymers, such as PEG or dextran. It is worth noting that dextran can be either linear or branched, depending on molecular weight and preparation. However, its physical structure, which can heavily impact experimental results, is seldom reported. The second is that most investigations only use one crowder at a time, while the cellular environment contains several crowding agents of various types and sizes. Therefore, more realistic experiments would include several crowders simultaneously. A third important area involves the connection between macromolecular crowding, liquid–liquid phase separation (LLPS), and the cellular environment. Macromolecular crowding has been shown to facilitate LLPS, which is at the basis of the formation of membraneless organelles within the cell. Furthermore, the environment inside these organelles is itself crowded, which likely dictates specialized dynamics. A clear understanding of the contribution of macromolecular crowding to LLPS formation and functionality is therefore critical.

## Figures and Tables

**Table 1:** Techniques used to study crowding.

Technique	Description
**Fluorescence Spectroscopy**	Fluorescent probes or fluorescently labeled macromolecules are used to monitor changes in the partitioning of proteins [13,14,15,16] or nucleic acids [17,18,19,20,21,22,23] in crowded environments.
**Dynamic Light Scattering (DLS)**	Fluctuations in the Rayleigh scattering of light due to diffusion and interference between particles in a solution can be used to assess changes in particle size and proximity in crowded conditions [24,25,26,27,28].
**Small-Angle X-ray Scattering (SAXS)**	The scattering of X-rays can be used on non-crystalline samples to determine the average size and shape of monodisperse macromolecules in crowded solutions [26,27,29].
**Nuclear Magnetic Resonance (NMR) Spectroscopy**	NMR can be used to monitor how crowding changes the electronic environment of nuclear spins in biomolecules and alters spin coupling [30,31,32].
**Electron Microscopy**	Electrons, which have very short wavelength with respect to photons, can be used to visualize, in vacuum conditions, metal-stained macromolecular structures prepared from dilute to crowded conditions with nanometer-scale resolution [8].
**Cryo-Electron Microscopy (Cryo-EM)**	Cryo-EM can be used to determine the structure of unstained macromolecular complexes held in tiny droplets of ice in crowded conditions at nanometer-scale resolution [33].
**Analytical Ultracentrifugation**	Optical detection of the dynamics and extent of migration of macromolecules through solutions of density gradients can be used to determine their sizes and reveal condensation in crowded environments [34,35].
**Steady-State and Time-Resolved Fluorescence Resonance Energy Transfer (FRET)**	The transfer of energy between natural or exogenous fluorphores in macromolecules can be used to measure distances between them and/or their labeled macromolecules in crowded environments [36,37].
**Gel Electrophoresis**	Electrophoretic migration of macromolecules through gel meshworks in dilute to crowded solutions can be used to reveal sizes and macromolecular associations [38,39].
**Single particle tracking, Tethered particle microscopy, Optical and Magnetic Tweezers**	Single particle tracking [40], Tethered particle motion (TPM) [17,41] or force spectroscopy with optical or magnetic tweezing (OT or MT) [17,42,43,44] can be used to reveal the dynamics of conformational changes in crowded environments.
**Atomic Force Microscopy (AFM)**	AFM imaging can be used to observe condensates resulting from the presence of crowders [45].
**Isothermal calorimetry**	ITC titrations are used to determine association constants, enthalpy and entropy of macromolecular interactions influenced by crowding [46].
**Circular Dichroism (CD)**	Circular dichroism spectroscopy can be used to study the conformation and association of biomolecules in crowded environments by analyzing their differential absorption of left- and right-circularly polarized light [27,46,47,48].
**Convex Lens-Induced Confinement**	Pressure from a convex lens can be used to isolate one or a few macromolecules in dilute to crowded solutions to study the conformation of and association between macromolecules [3].
**Molecular Dynamics Simulations**	Computer simulations can be used to model the behavior of macromolecules in crowded conditions and provide insights into their interactions and dynamics [31].

**Table 2:** Effects of crowding.

Effect of Crowding on DNA	Description/Effects
DNA Extension/Compaction	DNA can compact or extend depending on the conditions [47,49,50,51,52,53,54,55,56,57,58,59,60].
	Branched or rigid crowders may lead to greater compaction due to steric hindrance.
	Linear and flexible crowders may induce milder compaction. [2]
Thermal Stability	Macromolecular crowding has been shown to increase the thermal stability of DNA [46,61].
	Crowding agents can stabilize DNA structures and reduce denaturation [62,63].
DNA Configuration (Right/Left-Handed)	Specific conditions and crowders may favor transitions between right- and left-handed DNA helices [64].
Opening of ssDNA Hairpins	Crowding can impact the stability and kinetics of DNA secondary structures like hairpins, which are stabilized by crowding and open more slowly [22,23].
Protein Binding (Nucleoid-Associated)	Crowding can enhance the binding of proteins, such as nucleoid-associated proteins (NAPs), to DNA [41,49,51].
Transcription	Increased crowding has been shown to increase the efficiency of transcription initiation [37] and enhance the transcription rate [65].
Liquid–Liquid Phase Separation	Crowding can contribute to the phase separation of biomolecules, including DNA, leading to the formation of liquid condensates [14,15,43].
